# A Case Report of Post-COVID-19 Eosinophilic Enteritis: An Uncommon Diagnosis in an Adult

**DOI:** 10.7759/cureus.65265

**Published:** 2024-07-24

**Authors:** Jayasree Ravilla, Ping He, Anish V Patel

**Affiliations:** 1 Internal Medicine, Monmouth Medical Center, Long Branch, USA; 2 Gastroenterology, Rutgers Robert Wood Johnson Medical School, New Brunswick, USA

**Keywords:** endoscopy, jejunum, gastrointestinal, duodenum, eosinophilic enteritis

## Abstract

Eosinophilic esophagitis (EoE) and eosinophilic gastroenteritis (EGE), also known as eosinophilic enteritis (EoN), are both parts of the eosinophilic gastrointestinal disease (EGID) and share pathogenic similarities. Over the past two decades, the incidence and prevalence of EoE have rapidly increased, especially in Western countries, while EGE remains rare. Unlike EoE, no standard treatment strategies or guidelines have been established due to the extreme rarity of EGE, especially in Western countries. Here, we report a rare case of EoN in a 35-year-old female resulting from severe acute respiratory syndrome coronavirus 2 (SARS-CoV-2) infection.

## Introduction

Eosinophilic enteritis (EoN), also known as eosinophilic gastroenteritis (EGE), is a rare entity of primary eosinophilic gastrointestinal disease (EGID). The presence of intensive eosinophilic infiltrate on histopathology is the striking feature [1]. While EoN is most common in pediatric patients, adults can be affected as well, especially in the 3rd-5th decades of life. Without treatment, EoN rarely resolves spontaneously and often results in severe malabsorption leading to malnutrition. This case report discusses an adult presenting symptoms of acute gastrointestinal enteritis, including diarrhea, nausea, and vomiting, of unclear etiology. Subsequent laboratory investigations and endoscopic evaluation with biopsies confirmed eosinophilic infiltration, leading to the diagnosis of chronic enteritis of eosinophilic nature. This case points out the complex differential diagnostic process required to identify EoN, which necessitates a comprehensive understanding of both the clinical and histopathological features of the disease.

## Case presentation

A 35-year-old female with no significant medical history presented with abdominal pain, nausea, diarrhea, vomiting, and weakness that began three weeks ago. She reported that her vomitus and stool are non-bloody and non-bilious. She tested positive for COVID-19 two months prior to presentation but did not receive any treatment due to stable vitals. She denied any new complaints of chills, fever, hemoptysis, hematemesis, hematuria, hematochezia, chest pain, palpitations, or syncope. Her medications included ondansetron 4 mg oral tablet as needed for nausea and simethicone 125 mg daily as needed for abdominal discomfort. She had no known allergies and denied any recent travel history.

Her initial vitals were stable and physical examination revealed diffuse abdominal tenderness and +1 bilateral lower extremity edema. The basic labs on admission are shown in Table 1. An ultrasound abdomen which was done due to slightly elevated liver enzymes showed trace pericholecystic and right perinephric fluid without any cholelithiasis. A stool alpha-1 anti-trypsin was measured due to significantly low albumin which was 82 mL/day confirming protein-losing enteropathy. 

**Table 1:** Labs at the time of admission

Labs	Units	Value
White Blood Cells (WBC) (Reference: 4.5 to 11.0 × 10^3^/uL)	10^3^/uL	9.14
Hemoglobin (Reference: 12.1 to 15.1 g/dL)	g/dL	14.1
Hematocrit (Reference: 36 % - 44 %)	%	41
Platelets (Reference: 150 - 450 x 10^3^/uL)	10^3^/uL	253
Serum Sodium (Reference: 135 - 145 Mmol/L)	Mmol/L	140
Serum Chloride (Reference: 96-106 Mmol/L)	Mmol/L	111
Blood Urea Nitrogen (Reference: 6-24 mg/dL)	mg/dL	10
Serum Creatinine (Reference: 0.7-1.3 mg/dL)	mg/dL	0.8
Albumin (Reference: 3.4 - 5.4 g/dL)	g/dL	1.8
Alanine Transaminase (ALT) (Reference: 4-36 U/L)	U/L	64
Aspartate Transaminase (AST) (Reference: 8-33 U/L)	U/L	65
Alkaline Phosphatase (ALP) (Reference: 30-130 U/L)	U/L	32
Total Serum Bilirubin (Reference: 0.1-1.2 mg/dL)	mg/dL	0.4

**Figure 1:**
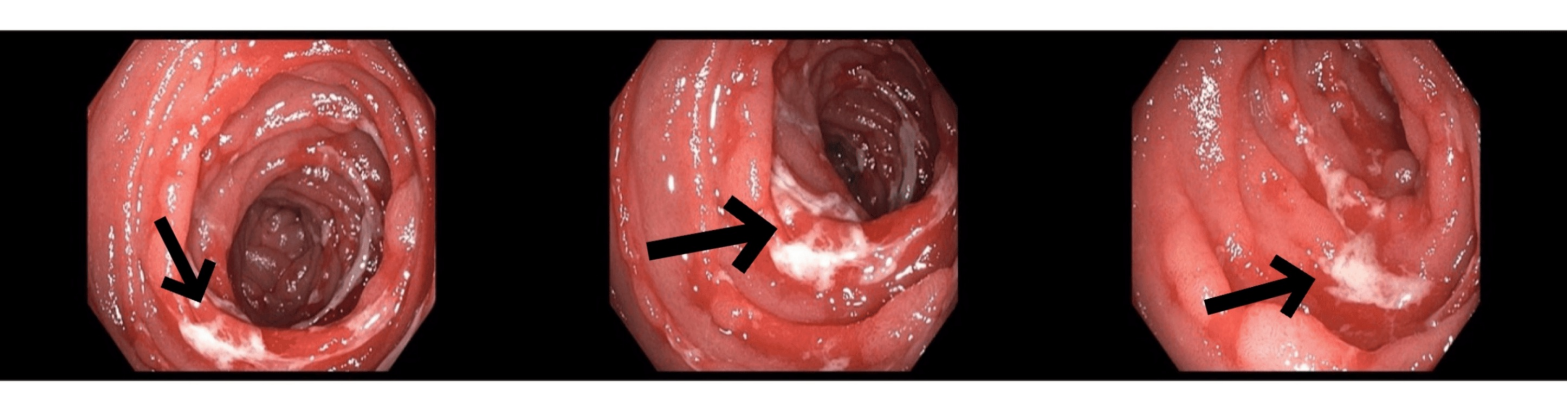
Erosion changes (arrows) suggestive of enteritis in the distal duodenum and proximal jejunum on EGD and enteroscopy EGD: Esophagogastroduodenoscopy

**Figure 2:**
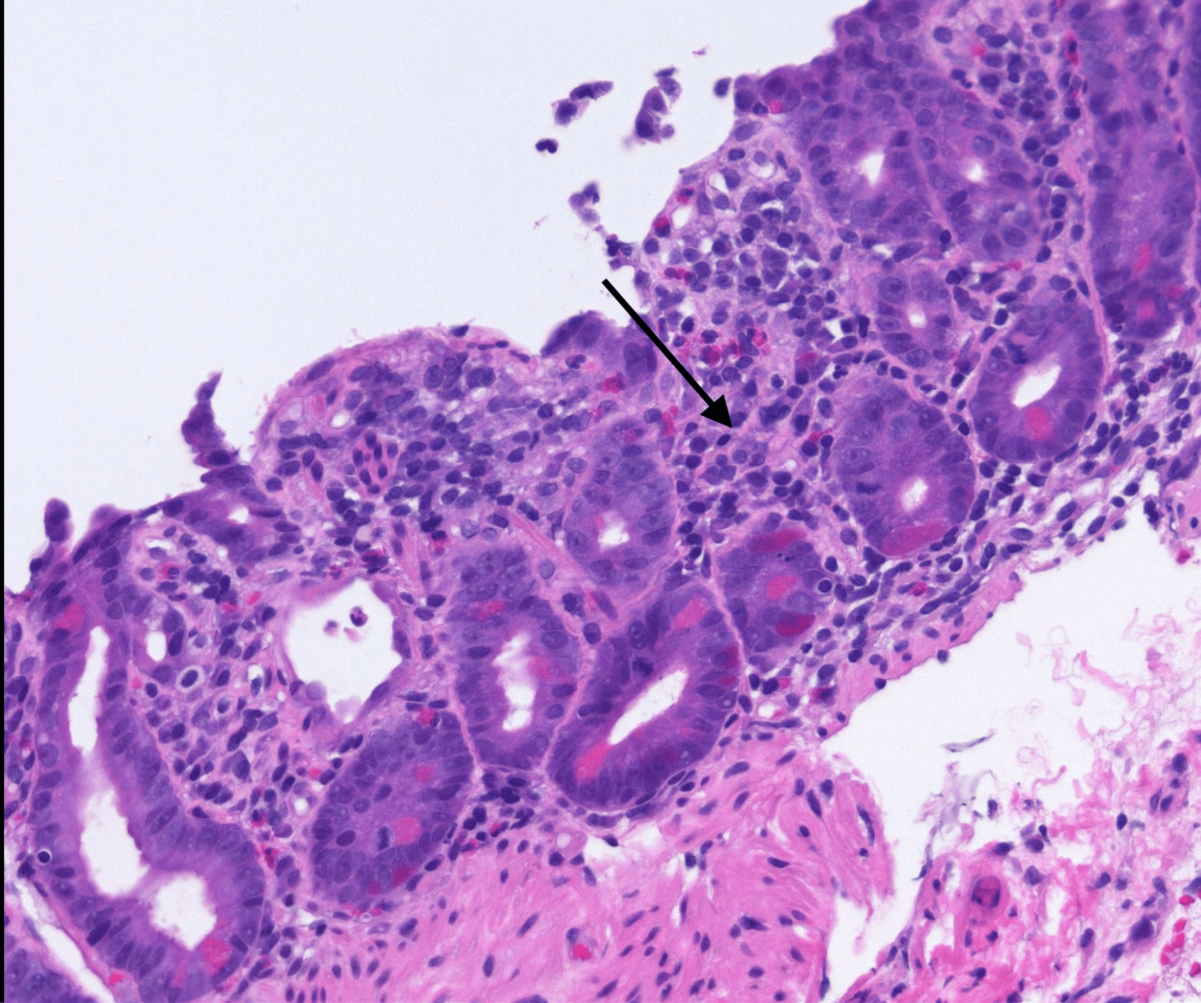
Patchy focal degranulation with eosinophilia on biopsies of the distal duodenum and proximal jejunum Black arrow showing eosinophilic infiltration on mucosal biopsy.

The other autoimmune panel including the celiac panel was negative (Table 2). Acute hepatitis viral panel and stool cultures were negative. Esophagogastroduodenoscopy (EGD) revealed diffuse erosions in the stomach and duodenum. Given the non-specific findings on EGD and persistent vomiting, a subsequent push enteroscopy was done which showed similar inflammatory changes in the jejunum (Figure 1). Histopathology reported up to 60 eosinophils per hpf in the lamina propria, with patchy focal degranulation, but no eosinophilic crypt abscess or infiltration into the muscularis mucosa (Figure 2). Colonoscopy along with biopsies was unremarkable. 

**Table 2:** Autoimmune panel

Lab	Value
Anti-smooth muscle antibody	Negative
Anti-mitochondrial antibody	Negative
Antinuclear antibody	Negative
Ceruloplasmin	Negative
Immunoglobulin G	Negative
Complement levels (C1, C2, C3, C4)	Negative
Anti-neutrophil cytoplasmic antibody	Negative
Fecal calprotectin	Normal

While inpatient, she was initially managed with furosemide 20 mg intravenous daily to improve edema. Pantoprazole 40 mg oral daily was started due to observation of erosions on EGD. Following the pathology report and suspected EoN, the patient was started on oral budesonide 3 mg daily for 14 days. A high-protein, medium-chain fatty acid-rich diet was recommended due to protein-losing enteropathy. A repeat surveillance EGD was done three months later which showed completely resolved erosions and absence of eosinophilia on histopathology. The trigger for EoN was unclear, but it was postulated it could be secondary to a recent COVID-19 infection that had preceded her hospitalization after excluding other possible causes of diarrhea and hypoproteinemia. Her symptoms were completely resolved in the follow-up visit. 

## Discussion

EGID is characterized by abnormal infiltration of eosinophils in the esophagus, stomach, small intestine, or colon, leading to organ dysfunction and a variety of clinical symptoms [1]. These diseases include eosinophilic esophagitis (EoE), eosinophilic gastritis (EG), EGE, EoN, and eosinophilic colitis (EC). Symptoms depend on the organs affected and the extent of infiltration of the intestinal wall [2,3].

Common symptoms of EoE are dysphagia and food impaction in adults, while children often suffer heartburn, abdominal pain, and vomiting. These disorders are immune-mediated chronic inflammatory conditions, which are linked to food allergen triggers [4]. The pathogenesis of EoN is believed to be a type 2 helper T-cell mediated immune response that leads to eosinophil chemotaxis and activation. The muscular form (in 12%) can cause intestinal strictures, abdominal pain, nausea, vomiting, and intestinal obstruction. The sub-serosal form (49%) is associated with eosinophilic-rich ascites bloating and abdominal pain [1]. There is a remarkable association between EoN and eosinophilia (>70%) and atopic disorders such as rhinitis, eczema, asthma, food and drug allergies, and dermatitis. Furthermore, 64% of patients have a family history of atopic diseases [5].

The diagnosis of EGID is based on the presence of gastrointestinal symptoms, biopsy showing eosinophilic infiltration in one or more areas of the gastrointestinal tract from the esophagus to the colon, and the absence of parasites or extraintestinal disease. These diseases are often associated with PLE because they cause severe diarrhea and increased gastrointestinal losses [6,7]. The histopathological criteria for the presence of eosinophils vary according to location. The criteria are as follows: EoE (>15 eosinophils/HPF), eosinophilic gastritis (>30 eosinophils/HPF), EoN (>30 eosinophils/HPF, >3 HPF), and EC (>25-65 eosinophils/HPF, depending on the colonic site) [8,9].

Treatment strategies are usually a combination of medication and dietary therapy aimed at controlling symptoms and intestinal inflammation. Dietary elimination therapy has limited efficacy, and topical steroids are less effective in treating EoE [2,4]. A variety of treatments have been shown to be effective, including dietary interventions, corticosteroids, mast cell stabilizers (e.g., sodium cromolyn), leukotriene receptor antagonists (e.g., montelukast), immunomodulators, biologic agents, and surgery [4,6]. Budesonide has a low intestinal absorption rate, is associated with fewer side effects than systemic steroids, and showed good results in our patients. EoN is common in pediatric patients but can also affect adults aged 30 to 50 years and, if left untreated, rarely resolves spontaneously and can lead to severe malabsorption and malnutrition. We could not find any association between COVID-19 infection and EoE in the literature. However, we were able to find an association with COVID-19 vaccination. This, along with our patient's timeline of presentation, helped us establish a link between EoE and COVID-19 infection [6,10,11].

## Conclusions

Although the exact etiology is not determined, there have been reports of EoN associated with the COVID-19 mRNA vaccine. Our case report highlights the possibility of enteritis occurring after the COVID-19 infection itself.
